# Integrating 4Ms Assessment through Medicare Annual Wellness Visits: Comparison of Quality Improvement Strategies in Primary Care Clinics

**DOI:** 10.3390/geriatrics8040070

**Published:** 2023-06-28

**Authors:** Sweta Tewary, Nicole Cook, Desiree Simon, Elizabeth Philippe, Oksana Shnayder, Naushira Pandya

**Affiliations:** 1 Geriatric Workforce Enhancement Program, Department of Geriatrics, Nova Southeastern University, Fort Lauderdale, FL 33328, USA; dsimon@nova.edu (D.S.); oshnayde@nova.edu (O.S.); pandya@nova.edu (N.P.); 2 OCHIN, Inc., Portland, OR 97228-5426, USA; cookn@ochin.org; 3 Brodes H. Hartley Jr. Teaching Health Center at Community Health of South Florida, Inc., 10300 SW 216th St., Miami, FL 33190, USA; ephilippe@chisouthfl.org

**Keywords:** age-friendly health system, annual wellness visit, older adults, health education, quality improvement

## Abstract

The Medicare Annual Wellness Visit (AWV), which includes comprehensive preventative assessments and screenings, is associated with improved preventative services, including vaccination and cancer screenings. However, the AWV alone does not promote whole-person care. Integrating the AWV within an Age-Friendly Health System (AFHS) contextualizes AWV services within a comprehensive geriatric care framework that integrates the “4Ms” (mentation, medication, mobility, and what matters). This study describes and evaluates quality improvement initiatives to improve the completion of AWV within two different AFHS-recognized health systems (an academic university clinic and a Federally Qualified Health Center). The results from this evaluation present opportunities that other health systems can consider for leveraging electronic health records (EHRs) and enabling services to complete AWVs within a 4Ms framework. The implementation results also suggest an adaptation of the 4Ms assessment schedule for patients with complex chronic conditions who may suffer from multiple comorbidities and cognitive impairment.

## 1. Introduction

### Background

The Medicare Annual Wellness Visit (AWV) is a recommended yearly appointment with a primary care provider that is conducted to create or update a comprehensive Personalized Prevention Plan (PPP). Medicare reimburses providers for one free AWV each year, including a review of medical and social history, counseling regarding preventative services, and the development of a PPP [1]. AWVs are associated with improved utilization and access to preventative services, including lifestyle behavior changes, vaccination, and cancer screenings [2]. Data also suggests that AWVs may be a potential revenue source for providers [3]. 

While AWVs have shown some benefits, the results are mixed [4]. There are well-documented barriers to completing AWVs as part of comprehensive care programs for older adults, including operational challenges, staffing, and technical issues [5]. In addition, AWVs have been criticized for not being tailored to meet the needs of geriatric patients with chronic disease as they do not incorporate several care elements related to functional status and patient needs [6]. Despite these challenges, AWVs are an important metric for healthcare organizations to achieve. HEDIS, the Healthcare Effectiveness Data and Information Set, collects data from healthcare organizations and uses the percentage of patients under each health plan who have completed the AWV as a measure of access to preventative and ambulatory healthcare services [7].

The Age-Friendly Health System (AFHS) approach provides a framework for integrating the components of the whole-person care approach into AWVs. The AFHS promotes whole-person health and well-being by assessing a patient’s current mentation status (utilizing dementia and depression screening); through appropriate medication management, including considering the deprescribing of unnecessary medications; via mobility testing (e.g., fall risk assessment); and by addressing what matters to the patient’s general well-being [8] as part of the care program. AFHS components are often referred to as the “4Ms” of care: mentation, medication, mobility, and what matters. In a previous paper, we described a training initiative for providers designed to improve the documentation of 4Ms during care [9]. The results of this initiative revealed that education alone did not lead to improvement in the 4Ms and suggested that alternative strategies may be needed to better integrate the 4Ms as part of AFHS care. This paper aims to compare and summarize two different Quality Improvement (QI) strategies to improve the quality of care for older adults by assessing the integration of the 4Ms into AWVs in two AFHS-recognized healthcare organizations.

## 2. Materials and Methods

### 2.1. Study Setting and Participants

We evaluated AWV QI strategies in two primary care clinics in South Florida that have both been designated as age-friendly clinics by the Institute of Healthcare Improvement (IHI). The University Clinic primarily serves geriatric patients with complex needs and is part of a medical-school-affiliated academic clinic in South Florida. The health center is a safety-net primary care clinic within a federally qualified health center serving largely medically vulnerable populations (low-income, racial/ethnic minorities). Study participants were older adults (aged 65 and above) enrolled in the clinic for primary care visits. Participants were selected based on convenience and accessibility in the clinics through convenience sampling. Prior to the development of the AWV QI projects, there was no specific program or policy in place to monitor the annual completion of AWVs at either of the clinics.

### 2.2. Overall Study Procedure

Both organizations received comprehensive training on AWVs, and AFHS offered in 2021 by the South Florida Geriatric Workforce Enhancement Program (SFGWEP), which is a member of a national network funded by the U.S. Department of Health and Human Services to train health professionals to meet the unique needs of ethnically and culturally diverse older adults. After being trained, both clinics independently decided to develop and implement a QI program to improve the percentage of eligible patients receiving AWVs incorporating 4Ms assessment. The implementation timeframe was three months for both organizations. 

### 2.3. Study Design

**Figure 1:**
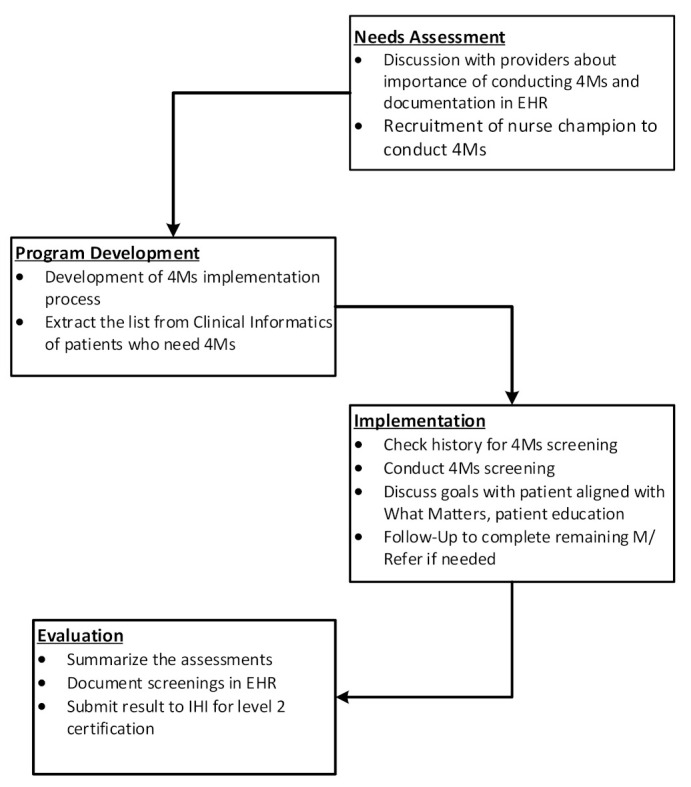
MRC Framework.

We used the non-sequential Medical Research Council Framework (MRCF) for the development and evaluation of the complex intervention [10]. The MRCF was used in our previous QI study to evaluate AFHS transformation [9]. This MCR framework guides evaluation through (1) feasibility/needs assessment, (2) program development, (3) implementation, and (4) evaluation. Figure 1 describes the framework’s stages and the activities we completed to facilitate the program. 

#### 2.3.1. Needs Assessment

University Clinic: The feasibility of QI implementation was evaluated via EHR chart review and through informal feedback from providers. EHR chart review was conducted by a nurse practitioner on 146 patient charts seen in three months from October to December 2021 to assess how the 4Ms were integrated into care for older adults, regardless of the status of the completion of the AWV. The chart reviews were focused on (1) mentation, which was measured through depression screening utilizing PHQ2 and cognition screening through MoCA, Mini-Cog, or MMSE as well as qualitative comments; (2) medication, measured through review and reconciliation; (3) mobility screening, which was assessed through two standardized questions regarding falls or injurious falls suffered during the past year, the Timed-Up-and-Go test (TUG), exercise, and physical therapy (PT) referral; and (4) ‘matters most’, which was measured through advance care planning (ACP) documentation. Informal feedback on challenges encountered during the completion of the 4Ms workflow process was provided by a nurse practitioner who provided AWV and 4Ms training in 2021 to providers. 

Health Center Clinic: The health center did not conduct a methodological needs assessment. The need for the QI initiative was determined by following the lists of patients who were outstanding with respect to receiving an AWV that were received from payors (e.g., managed care organizations) and a desire to further integrate AFHS 4Ms into older adult care. 

#### 2.3.2. QI Program Development

University Clinic: As part of program development and identifying the evidence base, the QI team discussed the strengths and limitations of the 4Ms implementation approach through face-to-face visits and telehealth. The team determined not to pursue telehealth due to limitations in assessing mobility and telehealth support at the patient and provider levels. The QI team, which included a nurse practitioner, a geriatrician, and an evaluator, developed a process flow (roadmap) that used an electronic health record (EHR) Medicare Preventative Template to facilitate the completion of AWV components. Under the QI program initiative, a dedicated nurse practitioner was assigned, one day a week for three months, to contact patients who were due or overdue for the AWV and schedule a face-to-face AWV, which was facilitated by the EHR Medicare Preventative Template. 

Health Center Clinic: The program development process at the health center clinic was conducted by a QI team including a geriatrician and a quality improvement and population health leadership team. Given the workforce shortages throughout the clinic and a reluctance to add additional workload to the providers, the QI team decided to involve medical residents from the health center to help complete AWVs for patients who were overdue according to the payor lists received by the clinic monthly. All residents had received AFHS and 4Ms training. Under the project plan, medical residents would receive a financial incentive to complete AWVs incorporating the 4Ms with patients on their own time (outside of residency rotation and didactic activities) using an EHR preventative services Smartform (e.g., EHR template) that integrates the 4Ms of AFHS. The QI team determined that due to ongoing COVID-19 precautions, the AWV initiative would be conducted through telehealth wherever/whenever it was feasible.

#### 2.3.3. Implementation/Testing of QI Program

University Clinic: The implementation process included the following activities conducted by a dedicated nurse practitioner who saw patients for their AWV: (1) asking older adults what matters most (e.g., with respect to health care goals, including advance care planning); (2) completing medication reviews and the identification of high-risk medications; (3) screening for cognitive impairment (Mini-Cog); (4) screening for depression (PHQ-2); and (5) screening for mobility limitations (TUG), guided by the EHR template. As part of the QI activities, the nurse practitioner implemented AFHS and 4Ms face-to-face with the help of rotating residents, students, and attending physicians from the clinic. This helped the clinic providers to share the workload.

Health Center Clinic: The implementation process in the health center clinic was led by medical residents assigned to ambulatory clinic rotations. This process supported provider access and the ability to record clinical data in the patient’s chart using the EHR AWV Smart form. The residents were assigned a list of patients due for their AWV and instructed to start with the patients due in December 2022. Residents contacted patients by phone. If patients could not be reached after the third attempt, a comment was made on the shared patient list to inform the care coordinator assigned to oversee the patient’s care. During the phone visits, providers either confirmed that the following 4Ms were completely implemented and documented in the chart during previous visits or completed the 4Ms assessment during the phone visit. If mobility was not recently addressed during in-person visits, it was assessed via phone visits by asking about recent falls. 

#### 2.3.4. Evaluation Process Design

Across both clinics, the evaluation included a review of progress regarding the completion of the documentation of patients’ 4Ms assessment using the EHR data.

University Clinic: Data on patients with whom an AMV had been completed during the three-month QI period were analyzed via EHR chart review by a nurse practitioner and recorded in an Excel spreadsheet with detailed information on medication reconciliation; depression screening utilizing PHQ2 and PHQ9; cognition assessment completed via MOCHA MiniCog or MMSE; mobility assessment completed via questions about falls and exercise and, if appropriate, a referral to Physical Therapy; and an assessment of what matters most completed by documenting the discussion of Advanced Care Planning or patient’s refusal to engage in this discussion. At the University Clinic, the project evaluator met with the clinic provider weekly to review any challenges regarding the completion of the 4Ms, including reminders for clinic providers. Additionally, students were engaged to help facilitate some of the 4Ms screening, including screenings for depression and dementia. 

Health Center Clinic: Ascertainment of AWV was determined via claims for AWV that had been successfully submitted after confirming the completion of the 4Ms by the QI team. We analyzed the data through retrospective chart reviews and descriptive statistics of 4Ms completion for all the patients in both organizations.

## 3. Results

### 3.1. Needs Assessment 

University Clinic: The needs assessment prior to the development of the QI project included both informal interviews with providers and an EHR chart review of 146 patients to ascertain how the 4Ms were historically integrated into the AWVs. The needs assessment identified challenges in relation to the complete implementation of the 4Ms including a lack of provider motivation to complete the 4Ms, time constraints, electronic health record (EHR) navigation, and technical issues with respect to documenting the 4Ms. Additional feedback from providers indicated that they experience difficulty in completely implementing the 4Ms due to the limited time during patient visits as a result of (1) there being too many complex chronic conditions or cognitive impairments to address; (2) questions and discourse with family members who attend visits with patients; (3) a lack of complete or updated medication lists; and (4) low literacy or language fluency. The EHR review of patient charts revealed that the providers were consistent with respect to completing medication reviews and reconciliation (146, 100%) but less consistent with respect to addressing mentation (41, 28%), mobility (57, 39%), and what matters most (20, 13.7%) for patients. Overall, only three patients (2%) had all 4Ms completed (See Table 1). The major challenge identified by providers was that the EHR assessments and screening workflow processes in the EHR were difficult to follow. The needs assessment was helpful in QI development, particularly in terms of recognizing the need for designated providers to complete AWVs consistent with the 4Ms. 

Health Center Clinic: No methodological needs assessments were conducted by the health center clinic’s QI team. The health center leadership held a planning meeting to strategize how the clinic could allocate scarce resources to increase the number of patients with AWVs following an identified need for the QI project. This meeting also included review of payor lists of patients with outstanding AWVs, and challenges associated with the existing workforce as a limiting factor in addressing 4Ms as part of AWV.

### 3.2. QI 4 Ms Implementation 

University Clinic: From 1 September 2022 to 1 December 2022, 15 patients had an AWV with a dedicated nurse practitioner. The breakdown of the 4Ms completed for the 15 patients is presented in Table 2. Complex patients who required multiple visits for the completion of the 4Ms were also flagged by the providers with reminders to be followed through at a later visit. 

Health Center Clinic: From 1 December 2022 to 1 March 2023, 22 patients were eligible for 4Ms screening, and 6 patients completed AWVs via phone call with residents (see Table 2). 

## 4. Discussion

The preliminary results from the implementation of a QI project at two different health care organizations suggest that dedicated team members helped to improve the completion of AWVs guided by the 4Ms and supported by EHR templates designed to promote AFHS. This finding may necessitate the modification of the clinic workflow to include geriatric assessments since these are traditionally not part of routine clinical care at many clinics. In our study, we found both approaches to completing 4Ms assessment in clinics effective. It was encouraging to see that assigning a 4Ms champion in clinical care expedited the 4Ms assessment. Incentivizing the providers also helped in completing the assessments. However, the results of the study should be interpreted with caution due to the possibility of selection bias impacting the internal validity of the study because of the use of convenience sampling.

At the university clinic, we learned that several factors may contribute to challenges in the completion of the 4Ms assessment as part of AWVs. The study highlighted difficulties in 4Ms completion for (1) patients with complex chronic conditions (e.g., six to nine comorbidities or cognitive impairment); (2) patients who come with family members, who sometimes require additional time for education; (3) patients who require a follow-up visit if the 4Ms assessments could not be completed in one visit; (4) patients who did not have complete or updated medication lists; and (5) patients with language issues. 

The challenges experienced by the health center clinic in completing AWVs and 4Ms assessment included the existence of some patients who could not be reached to schedule a visit. Therefore, it is unknown if patients due for AWVs expired or were subsequently assigned to an outside provider. For the health center clinic, it was not possible to fully evaluate mobility or the patient’s environment via a phone visit. Additionally, as many of the health center visits occurred after clinic hours, access to interpreter services for patients whose primary language was not English was not readily available. 

## 5. Conclusions

Completing the 4Ms assessment through AWVs helps to facilitate the completion of an overall physical, cognitive, and social assessment of older adults, which is often missed due to ongoing challenges in healthcare environments. Our QI projects evaluated two strategies for completing AWVs using the AFHS and 4Ms framework in two different organizations in south Florida. We found that both approaches supported AWVs and 4Ms completion but only with the dedicated providers’ time for assessment, which may have limited replicability without additional funding, resources, and support. 

Our paper adds to the body of knowledge concerning completing AWVs within an AFHS framework and provides two different approaches by describing two QI strategies. AWVs constitute one pathway to help complete 4Ms, and our paper describes the challenges of implementing QI programs in order to improve the completion rates of AWVs and 4Ms among adults at a university clinic and a health center. Future studies should also consider alternative methodologies for 4Ms completion. These could include intake forms completed by patients in waiting rooms, incorporating, and evaluating in-built EHR templates/smart forms, modifying workflows designed to support AWVs and 4Ms assessment, and evaluating the role of AWVs and 4Ms assessments in preventing the use of acute care services (e.g., emergency department and hospital visits and hospital readmissions).

## Figures and Tables

**Table 1:** University clinic needs assessment (retrospective chart review, *n* = 146).

AFHS “M” or Surrogate Addressed	No. Completed	% Completed
Medication—Review and reconciliation	146	100
Mentation—Depression or cognition addressed (PHQ2, qualitative comments mostly (few MOCHA, Mini-Cog, MMSE	41	28
Mobility addressed (falls questions, TUG test, exercise, PT referral)	57	39
Matters Most—ACP documentation or refusal	5	3.4
Medicare Preventive template completion (falls, function, PHQ2, mentation, ACP discussion)	20	13.7
All 4Ms were completed, including objective cognitive screening.	3	2

**Table 2:** QI Implementation.

AFHS “M” or Surrogate Addressed	No. (University Clinic)	% Completed	No. (Health Center)	% Completed
	Completed (15)		Completed (6)	
Medication Review and reconciliation	15	100	6	100
Mentation—Depression or cognition addressed (PHQ2, qualitative comments mostly (few MOCHA, Mini-Cog, MMSE	13	86.6%	6	100
Mobility addressed (falls questions, TUG test, exercise, PT referral)	15	100	6	100
Matters Most—ACP documentation or refusal	15	100	6	100
All 4Ms completed, including objective cognitive screening.	13	86.6	6	100

## Data Availability

Due to ethical, legal and privacy issues, data sharing is not applicable to this article.
